# Impact of Comorbid Hypertension and Diabetes on Anti‐VEGF Treatment Outcomes in Macular Edema

**DOI:** 10.1155/joph/8794635

**Published:** 2026-02-10

**Authors:** Weilin Lu, Shanshan Yang, Cong Zheng, Zhiyi Wu

**Affiliations:** ^1^ Department of Ophthalmology, Taizhou Municipal Hospital (Taizhou University Affiliated Municipal Hospital), School of Medicine, Taizhou University, Taizhou, 318000, China, tzc.edu.cn; ^2^ Department of Ophthalmology, Zhejiang University, Eye Center of Second Affiliated Hospital, School of Medicine, Hangzhou, 310009, China, zju.edu.cn

**Keywords:** anti-VEGF, best-corrected visual acuity, central macular thickness, diabetes, hypertension, macular edema

## Abstract

**Objective:**

This study evaluated the effects of intravitreal injection of antivascular endothelial growth factor (VEGF) treatment on visual acuity and macular edema severity in patients with comorbid hypertension and diabetes macular edema in China.

**Method:**

A longitudinal observational study, involving a total of 89 cases with macular edema who received anti‐VEGF injection treatment, compared the changes in visual acuity and macular edema severity among four groups of patients at three different time points. Additionally, through regression analysis, it explored the changes in best‐corrected visual acuity (BCVA) and central macular thickness (CMT) in patients with hypertension and diabetes after receiving different numbers of anti‐VEGF treatments, as well as the relationship between the number of injections and visual improvement and edema reduction.

**Results:**

Significant improvements in BCVA and CMT were observed following anti‐VEGF treatment, most notably after the first injection. The group without comorbidities demonstrated the greatest improvements, with BCVA improving from 0.20 to 0.40 logMAR and CMT decreasing from approximately 600 μm to 200 μm. In contrast, patients with hypertension and/or diabetes exhibited attenuated therapeutic responses. Multifactorial regression analysis confirmed that the presence of hypertension and/or diabetes served as an independent negative predictor for both BCVA improvement (*β* = 0.12, *p* = 0.002) and CMT reduction (*β* = −149.8, *p* < 0.001). Furthermore, poorer control of blood pressure and blood glucose levels was associated with diminished anatomical improvement.

**Conclusion:**

In summary, patients with both hypertension and diabetes face greater challenges in terms of their overall health condition, the rate of vision decline, and the improvement of macular edema. It is recommended to initiate treatment for patients with comorbidities earlier, increase the number of injections, and combine other therapies to enhance the treatment effect.

**Trial Registration:**

Taizhou Municipal Hospital: LWYJ2025268

## 1. Introduction

Macular edema is a prominent cause of vision loss, typified by retinal thickening in the central macular region due to fluid accumulation [1]. Patients often experience decreased visual acuity and metamorphopsia, which significantly hinder daily activities and visual function. The underlying pathogenesis involves disruption of the blood–retinal barrier (BRB), leading to the leakage of fluid and inflammatory factors into the extracellular space and initiating a cascade of pathological changes [2]. Macular edema can be categorized into several types, including diabetic macular edema (DME), retinal vein occlusion‐associated macular edema (RVO‐ME), and uveitic macular edema (UME) [3]. Although the etiologies of these subtypes differ, they are all closely associated with ocular vascular pathology or inflammatory responses. For instance, DME arises primarily from retinal microvascular changes induced by chronic hyperglycemia, which leads to BRB dysfunction [4]. RVO‐ME results from venous obstruction in the retina, impairing blood flow and increasing fluid leakage [5]. In contrast, UME is driven by intraocular inflammation that compromises the integrity of the BRB [6]. In clinical practice, many patients present with comorbid hypertension and diabetes, further complicated by macular edema [7]. This comorbidity is particularly challenging because both hypertension and diabetes can negatively impact ocular vasculature and exacerbate macular edema. Hypertension leads to thickening and sclerosis of the vascular walls, impairing blood flow, while diabetes induces microvascular changes. The combined effects of these two conditions render the BRB more susceptible to disruption, resulting in increased fluid leakage and macular thickening [8]. Therefore, for patients with this comorbidity, it is essential to actively treat macular edema and strictly control blood glucose and blood pressure levels to mitigate the progression of ocular pathology and preserve vision.

Anti‐VEGF agents have emerged as the cornerstone treatment for macular edema, achieving notable success in reducing retinal fluid accumulation and improving visual acuity [9]. However, it is essential to acknowledge that not all patients exhibit a robust response to anti‐VEGF therapy. For instance, patients with advanced diabetic retinopathy (DR) or chronic retinal vein occlusion (RVO) often fail to achieve significant improvements in vision or macular anatomy with anti‐VEGF monotherapy alone [10]. This limitation is partly attributed to the activation of the angiopoietin‐2/Tie2 pathway by chronic hypoxia and inflammation, which induces vascular leakage independent of VEGF and cannot be fully abrogated by anti‐VEGF agents alone [11]. Moreover, in patients with diabetes or hypertension, retinal pericyte apoptosis and thickening of the basement membrane can impede the repair of the BRB, resulting in recurrent fluid accumulation [12]. In RVO, the enlargement of the nonperfused area in the deep capillary plexus (DCP) leads to persistent high expression of VEGF, necessitating higher doses or combination therapies to achieve therapeutic efficacy [13]. Similarly, in DR, persistent leakage from microaneurysms is only partially inhibited by anti‐VEGF agents, underscoring the need for combination therapies to address the multifactorial nature of macular edema in these patients [14]. These findings underscore the complexity of macular edema and highlight the limitations of anti‐VEGF monotherapy in certain patient populations.

Presently, the majority of studies concentrate on the short‐term efficacy of anti‐VEGF therapy, typically evaluated within the first 3–6 months [15]. However, this limited time frame fails to capture the dynamic changes that occur over longer periods. A significant gap in the literature is the lack of long‐term follow‐up data extending beyond 1 year, which is essential for understanding the evolving patterns and sustained effects of treatment. To address this gap, our longitudinal observational study enrolled patients with macular edema who were undergoing anti‐VEGF therapy. We meticulously tracked the dynamic changes in BCVA using the LogMAR scoring system [16] and CMT over time [17]. By comparing these parameters across four distinct patient groups, we aimed to elucidate the temporal trends in BCVA and CMT changes. Additionally, we analyzed the relationship between the number of injections and therapeutic outcomes, providing valuable insights into the optimal treatment regimen. A novel aspect of our study is the first longitudinal investigation of the synergistic or antagonistic effects of hypertension and diabetes on the progression and treatment response of macular edema. Given the high prevalence of comorbid hypertension and diabetes among patients with macular edema, this analysis is crucial. By examining how these two common comorbidities interact and influence disease progression and treatment efficacy, our study aims to provide a foundation for personalized treatment strategies tailored to patients with multiple risk factors.

In summary, our research seeks to fill the gap in understanding the long‐term dynamics of macular edema treated with anti‐VEGF agents. By providing detailed insights into the temporal changes in visual and anatomical outcomes, as well as the impact of comorbidities, our study aims to contribute to the development of more effective and individualized treatment approaches for patients with macular edema.

## 2. Materials and Methods

### 2.1. Study Participants

A total of 89 patients with macular edema who underwent intravitreal anti‐VEGF injections were included in this longitudinal study. The sample size was estimated based on preliminary data from our institution, targeting a medium effect size (Cohen’s *f* = 0.25) for intergroup differences in BCVA change, with an alpha of 0.05 and a power of 0.80; the calculation indicated a minimum required total sample size of 68 eyes.

The study participants received either ranibizumab (manufactured by Novartis) or aflibercept (manufactured by Regeneron). The choice of agent was based on the treating physician’s discretion in accordance with Chinese clinical guidelines, drug availability, and patient‐specific factors such as disease etiology and prior treatment history. The treatment regimen (injection intervals and doses) followed a uniform “treat‐and‐extend” or “pro re nata” (PRN) protocol based on monthly monitoring across all groups, ensuring comparability. Patients were excluded if they had undergone systemic anti‐VEGF therapy, used intraocular corticosteroids, undergone vitreoretinal surgery, had significant anterior chamber opacity that prevented a detailed fundus examination, or had fewer than three follow‐up data points within the 6 months preceding the first injection. Demographic information (including blood pressure, blood glucose levels, age, and duration of vision loss) and ocular parameters were extracted from the patients’ medical records. The primary endpoints were BCVA, as assessed using the Snellen visual acuity chart, and CMT as a measure of edema severity.

### 2.2. Data Analysis

Statistical analyses were conducted utilizing GraphPad Prism software (GraphPad). To evaluate longitudinal changes in the measured parameters, we employed repeated‐measures analysis of variance (ANOVA) or mixed‐effects models, depending on the nature of the data. Post hoc analyses, which included pairwise comparisons, were carried out to determine differences at specific time points or within particular subgroups of the study population. The Wilcoxon signed‐rank test was used to analyze changes in BCVA and CMT before and after treatment. For comparisons of BCVA and CMT changes between different groups, the Mann–Whitney *U* test was applied. A *p*‐value of less than 0.05 was deemed to indicate statistical significance.

## 3. Results

### 3.1. Baseline Characteristics

This study included 89 patients with macular edema who received anti‐VEGF therapy. The participants were divided into four groups: 25 patients without hypertension or diabetes, 17 with only hypertension, 25 with only diabetes, and 22 with both hypertension and diabetes. The mean age of the patients was 60.7 years, with 46.1% (41/89) being males and 53.9% (48/89) being females. The diabetes‐only group had a slightly higher proportion of females. The group with hypertension and diabetes had the highest mean age at 64.2 years, which was significantly higher than that of the no comorbidity group (60.5 years; *p* = 0.046). There were no significant differences in height (143.0–177.0 cm) or weight (65.0–89.0 kg) among the four groups (*p* > 0.05). However, body mass index (BMI) trended higher in the hypertension group (24.9 kg/m^2^) and the hypertension and diabetes group (25.0 kg/m^2^) compared to the no hypertension and diabetes group (23.6 kg/m^2^, *p* = 0.073). At baseline, the diabetes‐only group had the worst LogMAR BCVA at 0.22, which was lower than that of the no comorbidity group (0.16) (*p* > 0.05). In the diabetes group and the hypertension and diabetes group, the proportion of DME patients was significantly higher than the other two groups (both > 70%), while in the no hypertension and diabetes group and the group with only hypertension, RVO‐ME was the main cause (Table 1).

**Table 1:** Patients’ baseline characteristics.

	No hypertension and diabetes (*n* = 25)	Hypertension (*n* = 17)	Diabetes (*n* = 25)	Hypertension and diabetes (*n* = 22)	*p* value
Male	14	11	15	11	
Female	11	6	10	11	
Age	60.5 (34–84)	65.6 (48–79)	58 (40–75)	64.2 (44–79)	0.046
Height	162.8 (150.0–177.0)	163.6 (150.0–175.0)	163.8 (150.0–174.0)	159.5 (143.0–173.0)	0.221
Weight	62.8 (45.0–76.0)	66.4 (55.0–80.0)	62.8 (53.0–80.0)	64.0 (48.0–89.0)	0.405
BMI	23.6 (18.7–30.0)	24.9 (19.5–30.7)	23.4 (19.5–26.7)	25.0 (19.6–30.8)	0.073
Baseline visual	0.16 (0.05–0.40)	0.16 (0.05–0.40)	0.20 (0.05–0.50)	0.22 (0.05–0.50)	0.304
Baseline blood pressure	135.4 (107–156)	143.6 (125–165)	134.0 (96–162)	154.2 (122–169)	< 0.001
	77.1 (58–98)	79.1 (67–98)	76.9 (56–99)	84.5 (65–102)	0.032
Baseline blood glucose	5.83 (4.06–8.30)	6.17 (5.10–9.60)	10.31 (4.60–25.90)	9.72 (3.70–20.90)	< 0.001
DME	0 (0.0)	0 (0.0)	18 (72.0)	16 (72.7)	
RVO‐ME	20 (80.0)	14 (82.4)	5 (20.0)	4 (18.2)	
UME	5 (20.0)	3 (17.6)	2 (8.0)	2 (9.1)	

### 3.2. Best‐Corrected Visual Acuity Progression

Among different patient groups, the mean BCVA exhibited significant improvement from the first to the third anti‐VEGF treatment session, while no significant differences were observed among subgroups with different injection timings. Specifically, in the group without hypertension or diabetes, BCVA significantly improved between the first and the second and third treatments (*p* < 0.05; Figure 1(a)), although no significant difference was found between the second and third treatments. In the hypertension‐only group (Figure 1(b)), a marginally significant difference was observed between the first and third treatments (*p* < 0.05), with no significant differences between the second treatment and the other two time points. In the diabetes‐only group (Figure 1(c)), a significant difference was noted between the first and third treatments (*p* < 0.05), but no significant differences were found in other pairwise comparisons. In the hypertension and diabetes group (Figure 1(d)), no significant differences were observed across all three treatment sessions. Regarding the impact of injection timing (Figures 1(e), 1(f), 1(g)), no statistically significant differences in BCVA were observed regardless of the presence of hypertension, diabetes, or both, in either the first or second injection.

Figure 1The changes in BCVA at different stages of anti‐VEGF treatment (data points represent mean ± SD at 1, 3, and 6 months postinitial injection). (a) The improvement of BCVA in the no hypertension and diabetes group. (b) The improvement of BCVA in the hypertension group. (c) The improvement of BCVA in the diabetes group. (d) The improvement of BCVA in the hypertension and diabetes group. (e) The differences in BCVA among each subgroup before the first injection. (f) The differences in BCVA among each subgroup before the second injection. (g) The differences in BCVA among each subgroup before the third injection.

**Figure:**
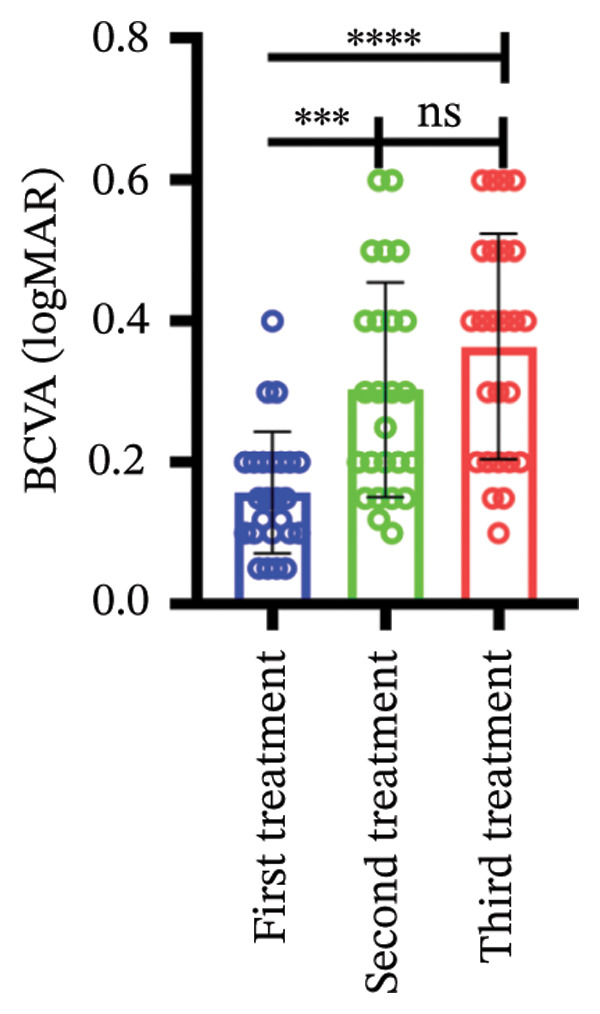
(a)

**Figure:**
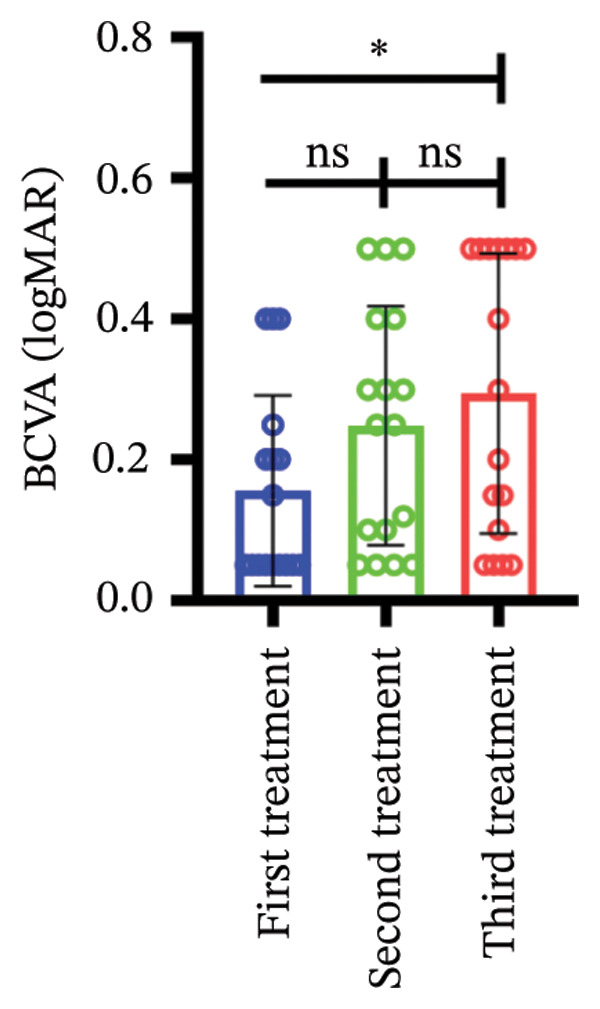
(b)

**Figure:**
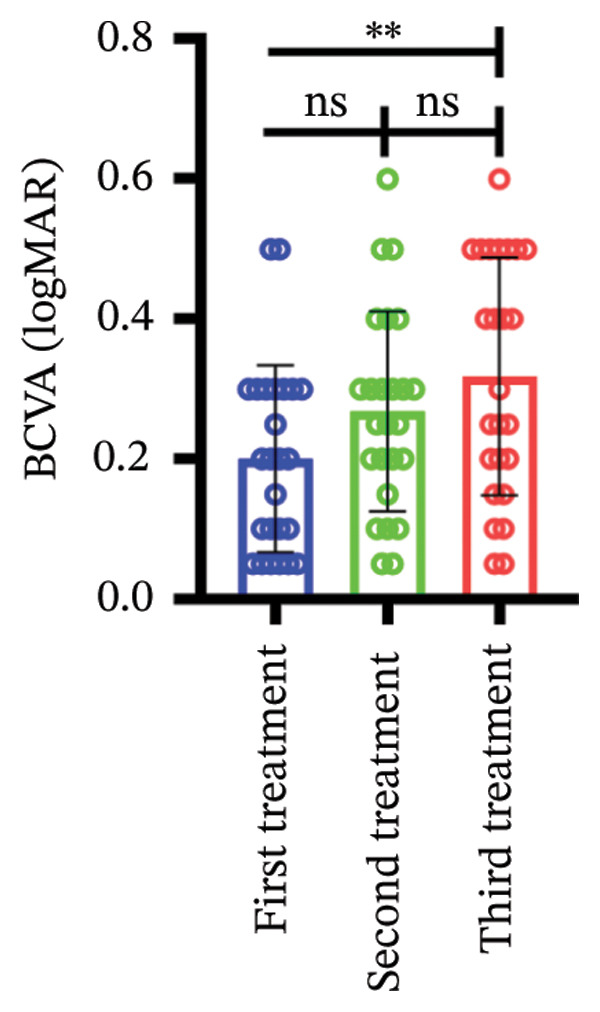
(c)

**Figure:**
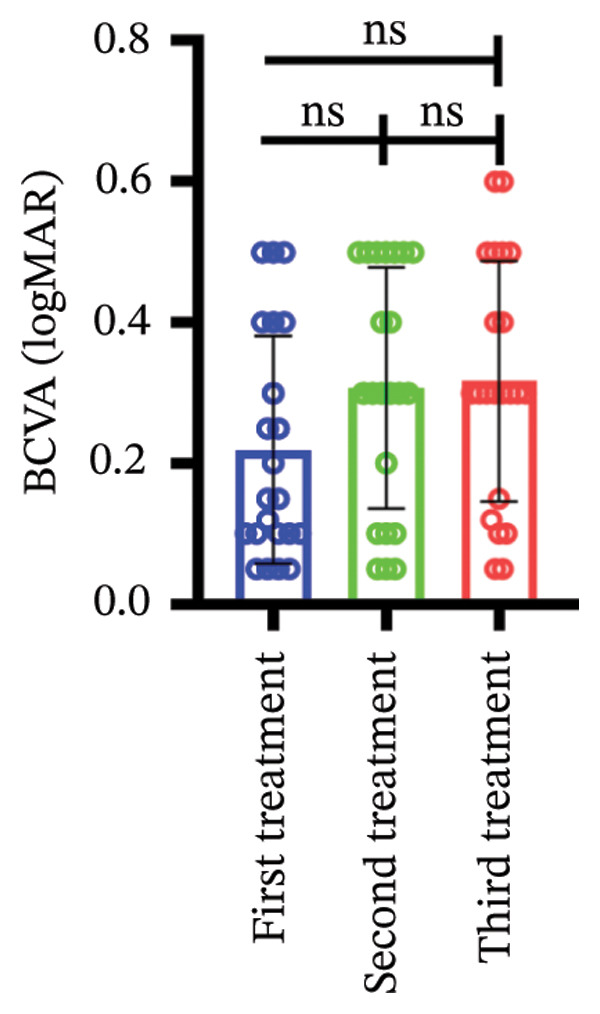
(d)

**Figure:**
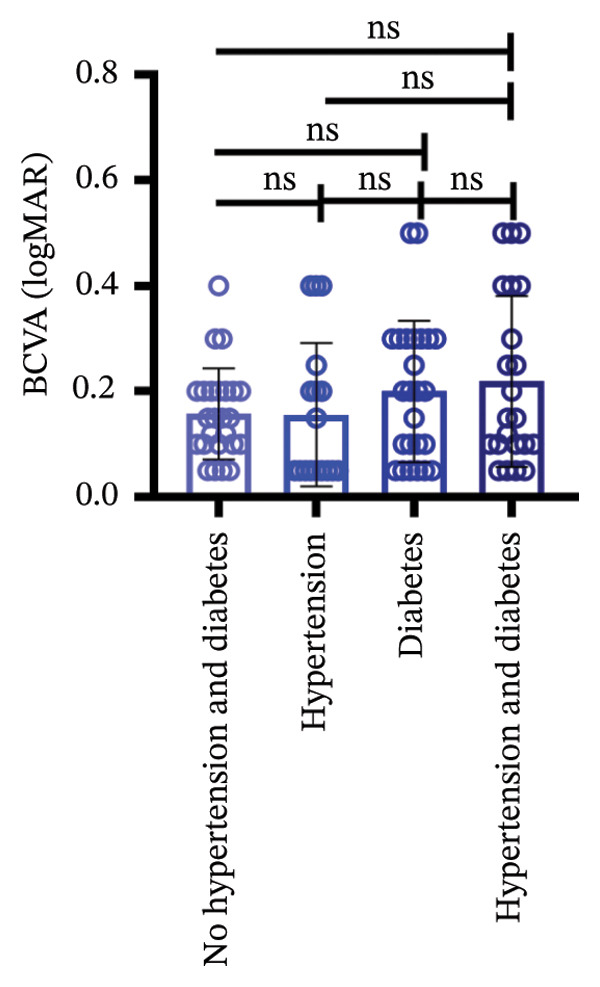
(e)

**Figure:**
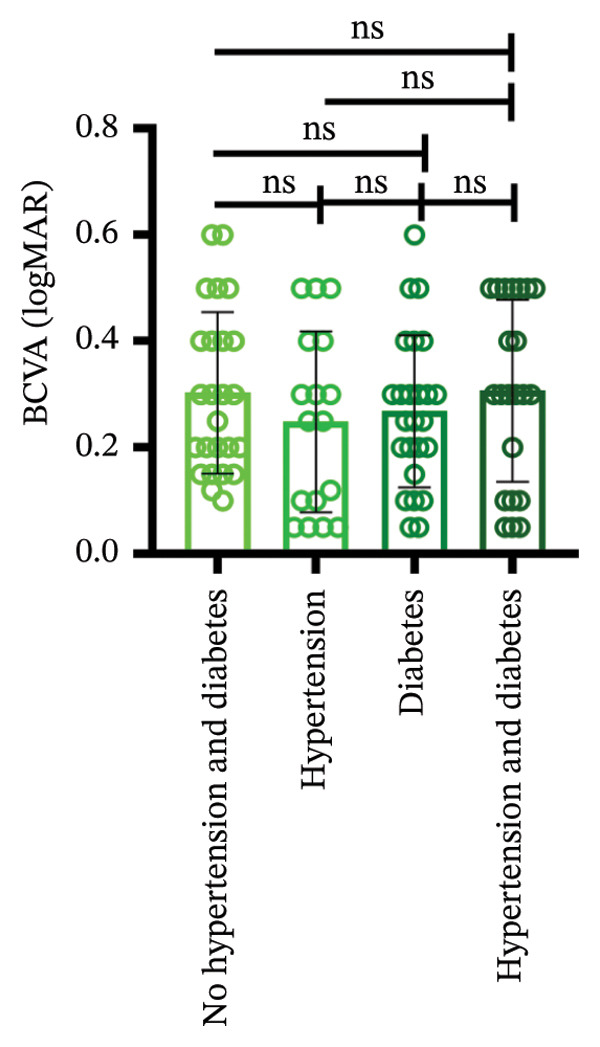
(f)

**Figure:**
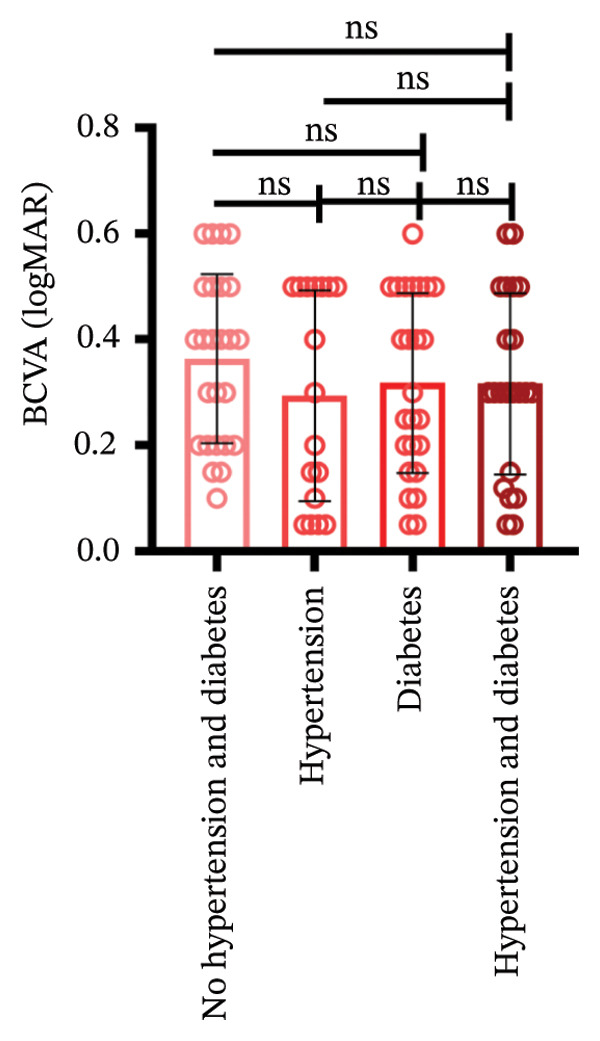
(g)

### 3.3. Central Macular Thickness (CMT) Progression

Following three treatment sessions, a significant decline in CMT was observed. In the cohort without hypertension or diabetes (Figure 2(a)), CMT was significantly reduced after the first treatment compared to the second (*p* < 0.05) and third treatments (*p* < 0.05), underscoring the pronounced effect of the initial treatment. No significant differences were detected between the second and third treatments. In the hypertension‐only group (Figure 2(b)), CMT remained relatively stable across all three treatments, with no statistical differences between the first treatment and the subsequent second or third treatments. This suggests that hypertension may attenuate the overall efficacy of the treatment. In the diabetes‐only group (Figure 2(c)), a significant decrease in CMT was noted after the first treatment (*p* < 0.05) compared to the second treatment, with no further improvements observed in subsequent treatments. No significant differences were found between the second and third treatments. In the group with comorbid hypertension and diabetes (Figure 2(d)), CMT also significantly decreased after the first treatment (*p* < 0.05) compared to the second treatment, with no changes in subsequent treatments. The overall reduction in CMT was intermediate between the hypertension‐only group and the healthy control group. No significant differences were observed between the second and third treatments. Prior to the first injection (Figure 2(e)), no significant differences in CMT were noted within the hypertension, diabetes, or combined hypertension and diabetes groups. However, significant differences were observed between the no hypertension and diabetes group and the other groups, as well as between the hypertension group and the combined hypertension and diabetes group. At the time of the second injection (Figure 2(f)), no significant differences in CMT were observed among the four groups. Before the third injection (Figure 2(g)), significant differences in CMT were noted between the no hypertension and diabetes group and the combined hypertension and diabetes group.

Figure 2The changes in CMT at different stages of anti‐VEGF treatment (data points represent mean ± SD at 1, 3, and 6 months postinitial injection). (a) The improvement of CMT in the no hypertension and diabetes group. (b) The improvement of CMT in the hypertension group. (c) The improvement of CMT in the diabetes group. (d) The improvement of CMT in the hypertension and diabetes group. (e) The differences in CMT among each subgroup before the first injection. (f) The differences in CMT among each subgroup before the second injection. (g) The differences in CMT among each subgroup before the third injection.

**Figure:**
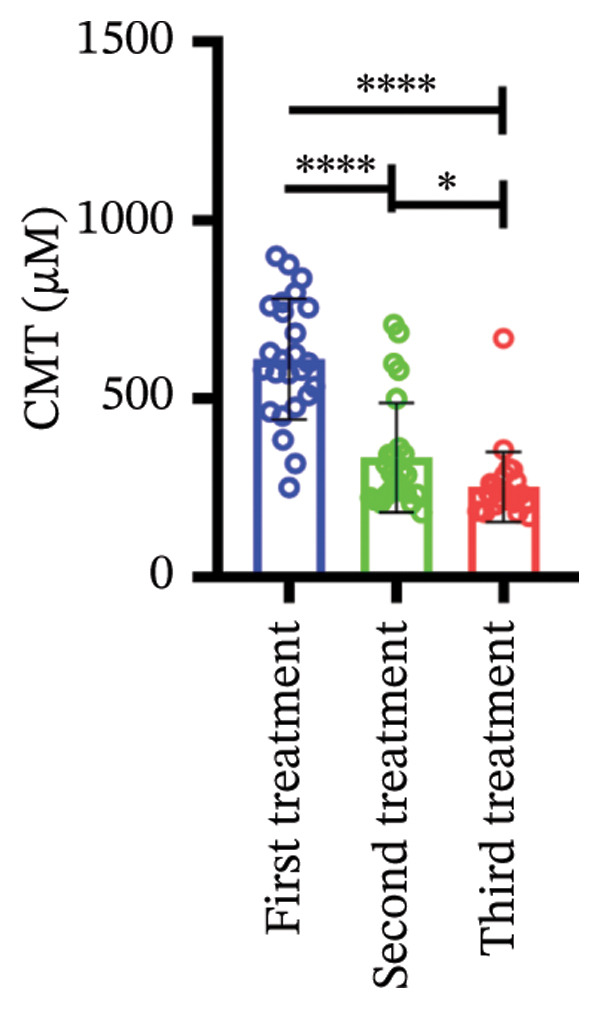
(a)

**Figure:**
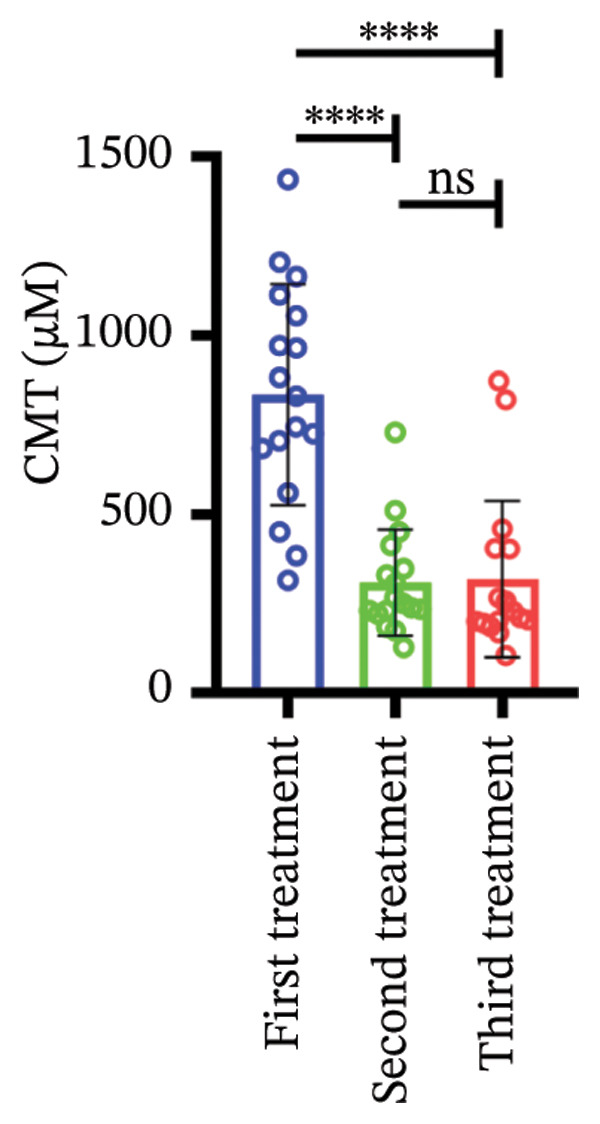
(b)

**Figure:**
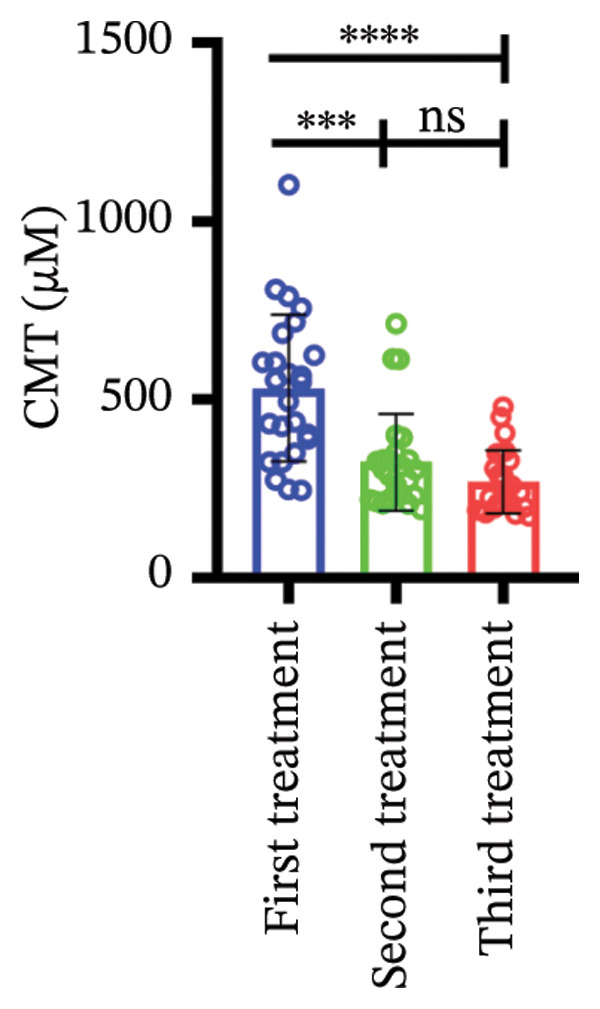
(c)

**Figure:**
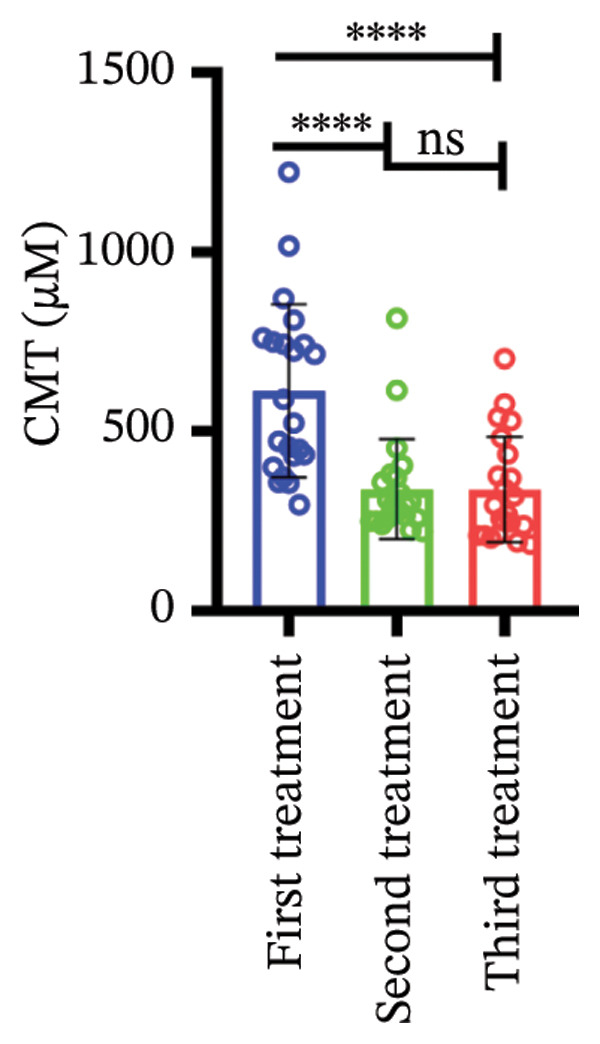
(d)

**Figure:**
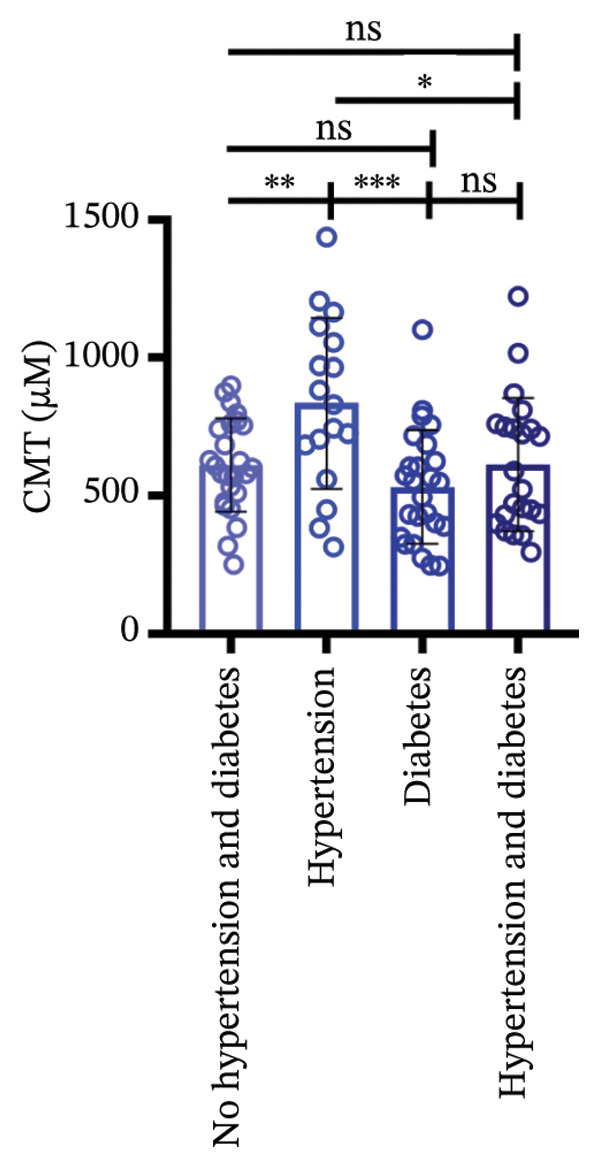
(e)

**Figure:**
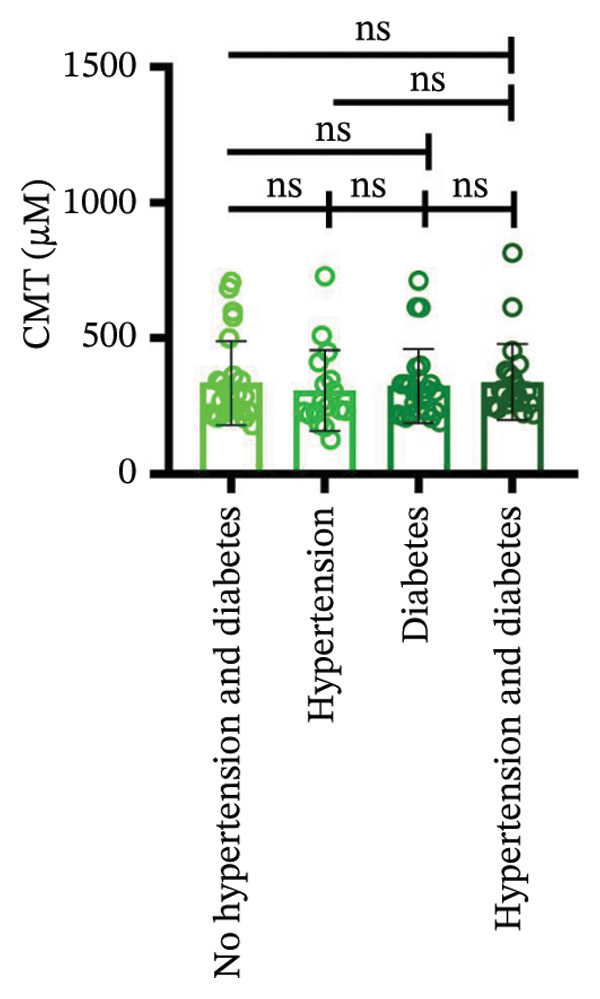
(f)

**Figure:**
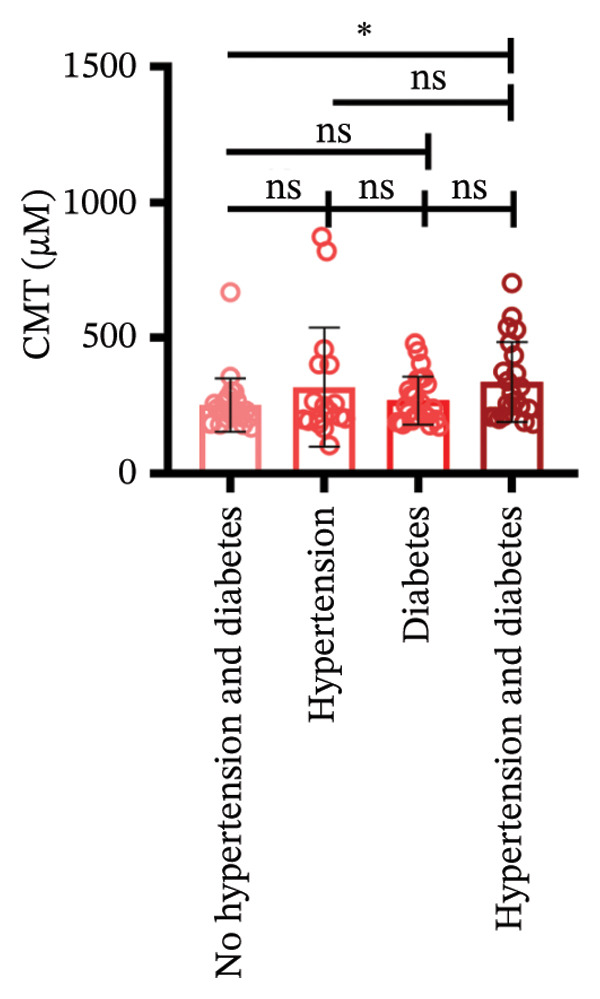
(g)

### 3.4. Changes in Best‐Corrected Visual Acuity and CMT Following Anti‐VEGF Treatments

The baseline BCVA was approximately 0.2 logMAR. After the first injection, BCVA significantly improved to around 0.3 logMAR. From the second to the third injection, BCVA remained relatively stable, hovering around 0.4 logMAR. Although the changes in BCVA appeared to plateau after the second injection, the overall improvement was statistically significant (Figure 3(a)). The CMT at baseline was approximately 600 μm. Following the first injection, CMT rapidly decreased to around 400 μm, indicating a substantial reduction in macular edema. With subsequent injections, CMT further decreased and stabilized at around 200 μm. The reduction in CMT was statistically significant, highlighting the efficacy of anti‐VEGF treatment in resolving macular edema (Figure 3(b)).

Figure 3The BCVA and CMT changes with the number of injections (mean ± SD). (a) BCVA increases with the increasing number of injections. (b) CMT decreases as the number of injections increases.

**Figure:**
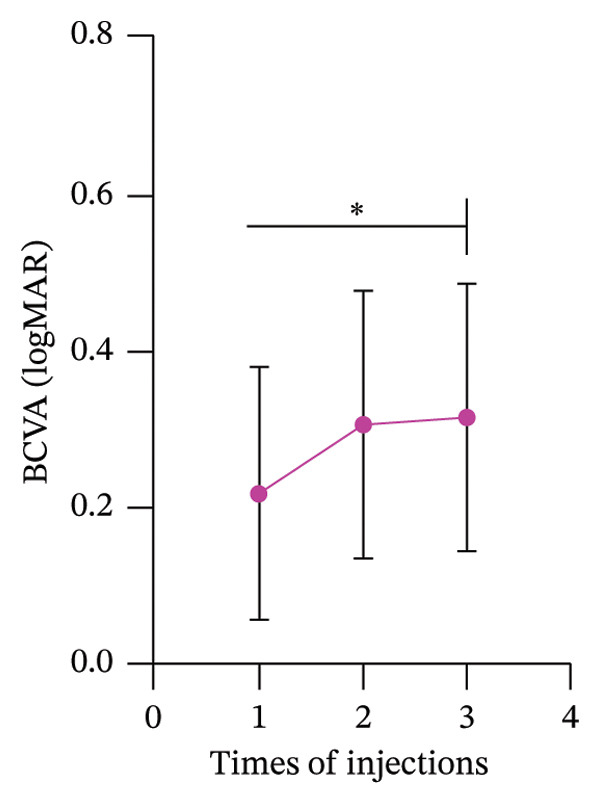
(a)

**Figure:**
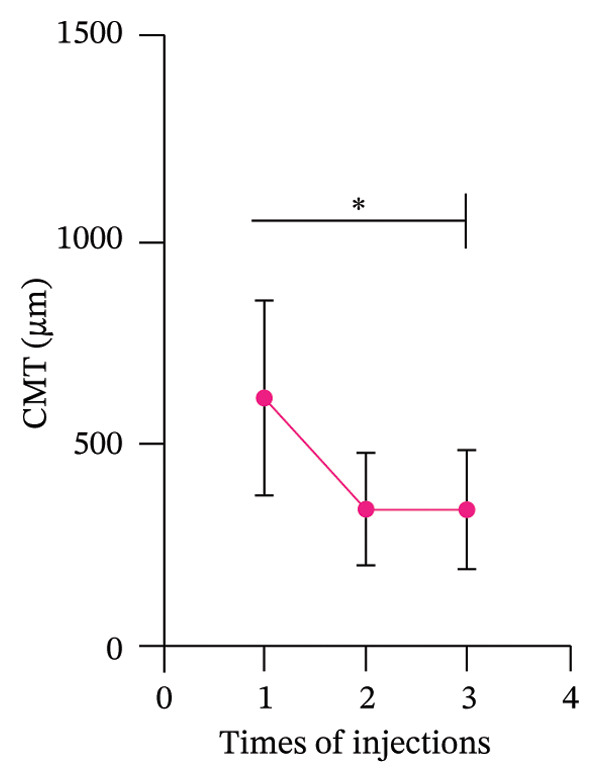
(b)

### 3.5. Multifactorial Analysis of Factors Influencing Treatment Efficacy

To elucidate the independent impact of comorbidities, a multifactorial linear regression analysis was performed. After adjusting for age, gender, baseline BCVA/CMT, and macular edema etiology, the presence of hypertension and/or diabetes remained a significant independent negative predictor for both functional and anatomical outcomes. Specifically, hypertension and diabetes group was associated with a lesser improvement in BCVA (*β* = 0.12, 95% CI: 0.05–0.20, *p* = 0.002) and a smaller reduction in CMT (*β* = −149.8, 95% CI: −226.1 to −73.5, *p* < 0.001) compared to the no hypertension and diabetes group (Table 2). Hypertension alone or diabetes alone also showed negative coefficients though with slightly lower significance levels.

**Table 2:** The results of the multivariate linear regression analysis of the factors influencing the improvement of BCVA and CMT.

Group	ΔBCVA (*β*, LogMAR)	*p* value	ΔBCVA (*β*, μm)	*p* value
Hypertension	0.05 (−0.02, 0.12)	0.166	−84.6 (−160.2, −9.0)	0.029
Diabetes	0.08 (0.01, 0.15)	0.025	−101.3 (−173.8, −28.8)	0.007
Hypertension and diabetes	0.12 (0.05, 0.20)	0.002	−149.8 (−226.1, −73.5)	< 0.001

### 3.6. Association of Blood Pressure and Glycemic Control With Treatment Outcomes

Analysis of systemic control metrics revealed that patients with poorer control had attenuated anatomical responses. The proportion of patients achieving target blood pressure control (< 140/90 mmHg) was 64.7% in the hypertension‐only group and 54.5% in the hypertension and diabetes group. Higher mean glucose levels correlated with less CMT reduction across all diabetic patients (*r* = 0.31, *p* = 0.042). Similarly, failure to achieve blood pressure targets was associated with a trend toward lesser BCVA improvement (*p* = 0.058).

## 4. Discussion

Macular edema frequently leads to vision loss and is especially common in conditions like DR and RVO. In hypertension, persistent hemodynamic stress and activation of the renin–angiotensin–aldosterone system (RAAS) lead to endothelial cell dysfunction, upregulation of proinflammatory cytokines, and increased oxidative stress, all contributing to chronic BRB leakage [18, 19]. In diabetes, hyperglycemia‐induced pathways including advanced glycation end products (AGEs) accumulation, protein kinase C (PKC) activation, and the polyol pathway cause pericyte apoptosis, basement membrane thickening, and sustained endothelial inflammation [20, 21]. These changes not only promote VEGF expression but also activate VEGF‐independent pathways that perpetuate vascular permeability. The retinal microvasculature in diabetes exhibits a reduced capacity for repair, making edema more refractory [22, 23]. Anti‐VEGF therapy has become a key treatment for macular edema, improving visual acuity by inhibiting VEGF activity, thereby reducing vascular leakage and macular edema [24]. In our longitudinal study, we administered anti‐VEGF treatment to patients with various clinical backgrounds and rigorously tracked changes in their visual acuity and macular edema over time.

Our investigation demonstrated that among patients with isolated macular edema, those who underwent two anti‐VEGF injections exhibited a marked enhancement in visual acuity, with additional improvements following a third injection. This highlights the effectiveness of early, standardized anti‐VEGF treatment and its cumulative impact on therapeutic outcomes. This finding is consistent with previous studies. For instance, Lanzetta P reported that early initiation of anti‐VEGF therapy significantly improves visual outcomes in patients with macular edema and that multiple injections can further consolidate these benefits [25]. These results suggest that in clinical practice, anti‐VEGF therapy should be initiated as early as possible and continued with multiple injections as indicated to achieve optimal visual outcomes [26].

Among individuals with isolated hypertension or diabetes, a significant enhancement in visual acuity was only observed following the third anti‐VEGF injection compared to the initial treatment. This delayed therapeutic response may be associated with fluctuations in blood pressure or blood glucose levels, which can modulate the effectiveness of anti‐VEGF agents [27]. Hypertension is frequently linked to abnormal vascular constriction, potentially impeding the penetration and efficacy of anti‐VEGF drugs [28]. Furthermore, oxidative stress induced by hyperglycemia can destabilize VEGF inhibitors, thereby reducing their therapeutic potency [29]. These observations are supported by prior research. For example, Ferrara N illustrated that vascular endothelial dysfunction in patients with hypertension and diabetes can profoundly affect the efficacy of anti‐VEGF treatment [30]. In our study, macular edema in patients with hypertension or diabetes decreased after the second injection but did not significantly change after the third. This stabilization may be attributed to the accelerated repair of macular leakage in hypertension and the persistent vascular endothelial damage caused by diabetes. These findings underscore the necessity for stringent control of blood pressure and glucose levels during treatment to mitigate their influence on therapeutic outcomes [31].

Patients with comorbid hypertension and diabetes, the least improvement in visual acuity was observed, with no significant differences noted within the group after three anti‐VEGF injections. This indicates that multiple comorbidities may significantly attenuate the cumulative efficacy of anti‐VEGF treatment [32]. In this specific subgroup, macular edema decreased only modestly after the second injection and exhibited minimal change after the third. The extent of macular edema remained significantly elevated compared to the normal group, suggesting that macular structural damage in patients with comorbidities is more refractory to treatment. This may require extended treatment duration and increased injection frequency to achieve optimal outcomes. These findings align with prior studies demonstrating that patients with multiple chronic conditions often experience poorer outcomes with anti‐VEGF therapy compared to those with a single disease [33]. In clinical practice, extending the treatment period, initiating anti‐VEGF therapy earlier, increasing injection frequency, and combining it with other modalities, such as laser photocoagulation or corticosteroid therapy, may be necessary to improve outcomes for patients with macular edema and comorbid hypertension and diabetes.

The first injection of anti‐VEGF treatment led to a notable improvement in BCVA and a significant reduction in CMT, thereby highlighting its immediate therapeutic efficacy. This rapid response is consistent with prior research indicating that anti‐VEGF agents can swiftly reduce vascular permeability and macular edema, ultimately enhancing visual acuity [34]. The substantial decrease in CMT from 600 μm to 200 μm represents a significant anatomical improvement in the macula, which is essential for long‐term visual outcomes. Given that persistent macular edema can result in irreversible structural damage and vision loss, the reduction in CMT is of utmost importance [35]. The stabilization of BCVA after the second injection and CMT at around 200 μm suggests that while further injections may not lead to dramatic improvements, they are crucial for maintaining the initial therapeutic gains. These results highlight the critical role of early anti‐VEGF intervention in patients with macular edema. The significant improvements observed after the first injection underscore the necessity of prompt treatment to prevent further vision loss and macular damage. Similar to previous reports, we observed that patients with combined hypertension and diabetes required more injections and exhibited slower anatomical recovery compared to those without comorbidities [36]. This aligns with the notion that systemic vascular and metabolic dysregulation contribute to a more refractory macular edema phenotype. In clinical practice, patients should be closely monitored following the initial injection, with additional injections administered as needed to sustain therapeutic efficacy. The stabilization of BCVA and CMT indicates that further injections, although not yielding additional gains, are essential for the long‐term management of macular edema. Patients with comorbid hypertension and diabetes often exhibit more refractory macular structural damage, necessitating longer treatment durations and more frequent injections to achieve better outcomes. Previous studies have demonstrated that patients with multiple chronic diseases tend to have worse outcomes with anti‐VEGF treatment compared to those with a single disease [37]. This resonates with mechanistic insights linking systemic inflammation and endothelial dysfunction to refractory edema [38]. Similarly, the benefit of extended treatment duration and combination therapies in high‐risk groups mirrors recommendations from large cohort studies [39]. Therefore, for patients with macular edema and comorbid hypertension and diabetes, extending the treatment period, initiating anti‐VEGF therapy earlier, increasing the number of injections, and combining it with other therapies, such as laser photocoagulation or corticosteroid treatment, may be necessary to improve therapeutic outcomes. Additionally, a positive correlation was observed between the number of injections and visual acuity improvement. However, the slope of improvement was lower in the group with hypertension and diabetes, indicating that more injections are needed for this group to achieve the same therapeutic effect as the normal group. Each additional injection led to a greater reduction in macular edema, further emphasizing the importance of tailoring the number of injections to the specific circumstances of each patient during the treatment process. Previous studies have also demonstrated that multiple injections can effectively improve vision and macular structure in patients with macular edema [40]. It is important to note that multiple injections may increase treatment costs and patient discomfort. Therefore, in clinical practice, personalized treatment plans should be developed based on a careful weighing of the pros and cons.

In conclusion, patients with comorbid hypertension and diabetes exhibit significantly poorer outcomes when treated with anti‐VEGF therapy, requiring prolonged and intensified treatment regimens to achieve optimal results. These observations provide valuable guidance for clinical practice, highlighting the need for a more aggressive and comprehensive treatment strategy for this high‐risk patient group. However, several limitations of the current study should be acknowledged. The relatively short follow‐up duration of 6 months precludes assessment of long‐term treatment efficacy, durability of response, and recurrence risk of macular edema, which are critical for understanding the chronic management needs of these patients. The exclusion of other systemic comorbidities known to affect retinal vasculature, such as dyslipidemia, chronic kidney disease, and autoimmune disorders, may limit the generalizability of our findings to real‐world populations with multimorbidity. Detailed data on treatment adherence and strictness of injection intervals were not available, which could influence both anatomical and functional outcomes and confound the interpretation of dosing‐frequency effects. Additionally, the modest sample size may limit the robustness and broader applicability of the results. As a single‐center study, it may also be susceptible to biases related to geographic and demographic factors. Furthermore, the study did not comprehensively track and analyze the control of blood pressure and blood glucose levels over the treatment period, which could impact the interpretation of the findings regarding systemic management. Future research should focus on increasing the sample size, conducting multicenter trials with extended follow‐up, incorporating a broader range of systemic comorbidities, rigorously documenting treatment adherence, and thoroughly monitoring the management of comorbidities to more accurately evaluate the efficacy and determinants of anti‐VEGF treatment in various clinical contexts.

## 5. Conclusion

This investigation explores how hypertension and diabetes influence the effectiveness of anti‐VEGF treatment for macular edema. Individuals with both hypertension and diabetes had worse initial health profiles and experienced more rapid vision decline, indicating that these conditions may work together to worsen retinal harm. Anti‐VEGF therapy started early helped improve vision and reduce macular thickness in patients without other issues, but its benefits were lessened in those with hypertension or diabetes. Hypertension made it harder for the drug to reach its target due to blood vessel problems, while diabetes‐related oxidative stress weakened the stability of VEGF inhibitors, resulting in less effective edema reduction. Importantly, patients with both conditions needed three injections to see any improvement, yet still had lingering structural issues, highlighting their resilience to standard treatments. These results suggest that tailored treatment plans, including longer treatment periods, earlier start times, and combined therapies targeting both vascular and inflammatory pathways, are needed to optimize outcomes.

**Nomenclature**
VEGF: vascular endothelial growth factor
BCVA: best‐corrected visual acuity
CMT: central macular thickness
DME: diabetic macular edema
RVO‐ME: retinal vein occlusion‐associated macular edema
UME: uveitic macular edema
BRB: blood–retinal barrier
RVO: retinal vein occlusion
DCP: deep capillary plexus
DR: diabetic retinopathy
BMI: body mass index
PRN: pro re nata
RAAS: renin–angiotensin–aldosterone system
AGEs: advanced glycation end products
PKC: protein kinase C

## Author Contributions

Weilin Lu: conceptualization, writing–original draft, investigation, analysis, and resources. Shanshan Yang and Cong Zheng: analysis and resources. Zhiyi Wu: supervision, conceptualization, methodology, and writing–review and editing.

## Funding

The work was not funded by any funding.

## Disclosure

All authors have accepted responsibility for the entire content of this manuscript and approved its submission.

## Ethics Statement

The study was conducted in accordance with the principles of the Declaration of Helsinki. All participants were fully informed about the nature, purpose, and potential risks of the study, and their consent was freely given without any coercion or undue influence. Additionally, the study protocol was reviewed and approved by the Taizhou Municipal Hospital, ensuring that the rights and well‐being of the participants were protected throughout the research process.

## Consent

Please see the Ethics Statement.

## Conflicts of Interest

The authors declare no conflicts of interest.

## Data Availability

The experimental data used to support the findings of this study are available from the corresponding author upon request.
